# Use and Usefulness of Dynamic Face Stimuli for Face Perception Studies—a Review of Behavioral Findings and Methodology

**DOI:** 10.3389/fpsyg.2018.01355

**Published:** 2018-08-03

**Authors:** Katharina Dobs, Isabelle Bülthoff, Johannes Schultz

**Affiliations:** ^1^Department of Brain and Cognitive Sciences, Massachusetts Institute of Technology, Cambridge, MA, United States; ^2^Department Human Perception, Cognition and Action, Max Planck Institute for Biological Cybernetics, Tübingen, Germany; ^3^Division of Medical Psychology and Department of Psychiatry, University of Bonn, Bonn, Germany

**Keywords:** dynamic faces, facial animation, facial motion, dynamic face stimuli, face perception, social perception, identity-from-motion, facial expressions

## Abstract

Faces that move contain rich information about facial form, such as facial features and their configuration, alongside the motion of those features. During social interactions, humans constantly decode and integrate these cues. To fully understand human face perception, it is important to investigate what information dynamic faces convey and how the human visual system extracts and processes information from this visual input. However, partly due to the difficulty of designing well-controlled dynamic face stimuli, many face perception studies still rely on static faces as stimuli. Here, we focus on evidence demonstrating the usefulness of dynamic faces as stimuli, and evaluate different types of dynamic face stimuli to study face perception. Studies based on dynamic face stimuli revealed a high sensitivity of the human visual system to natural facial motion and consistently reported dynamic advantages when static face information is insufficient for the task. These findings support the hypothesis that the human perceptual system integrates sensory cues for robust perception. In the present paper, we review the different types of dynamic face stimuli used in these studies, and assess their usefulness for several research questions. Natural videos of faces are ecological stimuli but provide limited control of facial form and motion. Point-light faces allow for good control of facial motion but are highly unnatural. Image-based morphing is a way to achieve control over facial motion while preserving the natural facial form. Synthetic facial animations allow separation of facial form and motion to study aspects such as identity-from-motion. While synthetic faces are less natural than videos of faces, recent advances in photo-realistic rendering may close this gap and provide naturalistic stimuli with full control over facial motion. We believe that many open questions, such as what dynamic advantages exist beyond emotion and identity recognition and which dynamic aspects drive these advantages, can be addressed adequately with different types of stimuli and will improve our understanding of face perception in more ecological settings.

## Introduction

Most faces we encounter and interact with move - when we meet a friend, we display continuous facial movements such as nodding, smiling and speaking. From the information conveyed by dynamic faces, we can extract cues about a person's state of mind (e.g., subtle or conversational facial expressions; Ambadar et al., 2005; Kaulard et al., 2012), about their focus of attention (e.g., gaze motion: Emery, 2000; Nummenmaa and Calder, 2009), and about what they are saying (e.g., lip movements; Rosenblum et al., 1996; Ross et al., 2007). Despite this extensive information conveyed by dynamic faces, much of it is already contained in their static counterpart, including sex, age or basic emotions (Ekman and Friesen, 1976; Russell, 1994). Therefore, and for ease of use, most face perception studies rely on static stimuli. When do dynamic faces provide additional information to static faces, and what is this information? What kind of stimuli is appropriate to study different aspects of dynamic face perception? In this review, we will discuss findings on the usefulness of dynamic faces to study face perception, followed by an overview of methodological aspects of this work. We conclude with a brief discussion, future directions and open questions.

### Human sensitivity to spatio-temporal information in dynamic faces

Before designing any study using dynamic faces, it seems relevant to ask how sensitive the human visual system is to facial motion. Are simple approximations sufficient, or is the face perception system finely attuned to natural motion? Recent evidence supports the latter: In a recent study, we systematically manipulated the spatio-temporal information contained in animations based on natural facial motion (Dobs et al., 2014). Subjects chose in a delayed matching-to-sample task which of two manipulated animations was more similar to natural motion. Subjects consistently selected the animations closer to natural motion, demonstrating high sensitivity to deviations from natural motion. In line with these results, face stimuli based on motion created by linear morphing techniques (e.g., linear morphing between two frames) can lead to less accurate emotion recognition (Wallraven et al., 2008; Cosker et al., 2010; Korolkova, 2018) and are often perceived as less natural (Cosker et al., 2010) than natural motion. Moreover, humans are sensitive to specific properties of natural motion (e.g., velocity; Pollick et al., 2003; Hill et al., 2005; Bould et al., 2008), to temporal sequencing (e.g., temporal asymmetries in the unfolding of facial expressions; Cunningham and Wallraven, 2009; Reinl and Bartels, 2015; Delis et al., 2016; Korolkova, 2018) and even to perceptual interactions between dynamic facial features (e.g., eye and mouth moving together during yawning; Cook et al., 2015). Given this high sensitivity, what is the additional value of facial motion?

### Is there an added value of dynamic compared to static faces?

It seems intuitive to assume that dynamic information (e.g., a video) would facilitate the identification of facial expressions compared to static images (dynamic advantage), because expressions develop over time. However, this assumption is subject to some controversy (Krumhuber et al., 2013). Most studies report a dynamic advantage for expression recognition (Harwood et al., 1999; Ambadar et al., 2005; Bould et al., 2008; Kätsyri and Sams, 2008; Cunningham and Wallraven, 2009; Horstmann and Ansorge, 2009; Calvo et al., 2016 (for synthetic faces); Wehrle et al., 2000), while others do not (Jiang et al., 2014 (under time pressure); (Widen and Russell, 2015) (for children); (Kätsyri and Sams, 2008) (for real faces); Fiorentini and Viviani, 2011; Gold et al., 2013; Hoffmann et al., 2013).

This controversy might have arisen from differences in stimuli and paradigms or from the methods used to equalize the stimuli (Fiorentini and Viviani, 2011). For example, most studies reporting a lack of a dynamic advantage have tested basic emotions and compared the expression's peak frame as static stimulus against the video sequence (e.g., Kätsyri and Sams, 2008; Fiorentini and Viviani, 2011; Gold et al., 2013; Hoffmann et al., 2013). In contrast, in studies reporting a dynamic advantage, either the authors presented degraded or attenuated basic emotion stimuli (Bassili, 1978; see also Bruce and Valentine, 1988; but see Gold et al., 2013) or observers had difficulty extracting information from the stimuli (for example, autistic children and adults: Gepner et al., 2001; Tardif et al., 2006; but see Back et al., 2007); individuals with prosopagnosia: (Richoz et al., 2015), or more complex and subtle facial expressions were tested (Cunningham et al., 2004; Cunningham and Wallraven, 2009; Yitzhak et al., 2018). These findings suggest that the dynamic advantage is stronger for subtle than basic expressions, while a dynamic advantage for basic emotions can be best observed under suboptimal conditions (Kätsyri and Sams, 2008).

### Perception of dynamic face information beyond emotional expressions

Facial motion does not only enhance facial expression understanding, but can also improve the perception of other face aspects. For example, one robust finding is that facial motion enhances speech comprehension when hearing is impaired (Bernstein et al., 2000; Rosenblum et al., 2002). Facial motion also conveys cues about a person's gender (Hill and Johnston, 2001) and identity (Hill and Johnston, 2001; O'Toole et al., 2002; Knappmeyer et al., 2003; Lander and Bruce, 2003; Lander and Chuang, 2005; Girges et al., 2015). Interestingly, the amount of identity information contained in facial movements depends on the type of facial movement: In a recent study (Dobs et al., 2016), we recorded from several actors three types of facial movements: emotional expressions (e.g., happiness), emotional expressions in social interaction (e.g., laughing with a friend), and conversational expressions (e.g., introducing oneself). Using a single avatar head animated with these facial movements, we found that subjects could better match actor identities based on conversational compared to emotional facial movements. Importantly, ideal observer analyses revealed that conversational movements contained more identity information, suggesting that humans move their face more idiosyncratically when in a conversation. Similar to the dynamic advantage for facial expressions, these findings show that the visual system can use identity cues in facial motion when form information is degraded or absent. However, whether this phenomenon occurs in real life in the presence of identity cues carried by facial form was still unclear (O'Toole et al., 2002). In a recent study (Dobs et al., 2017), we systematically modified the amount of identity information contained in facial form versus motion while subjects performed an identity categorization task. Based on optimal integration models, we showed that subjects integrated facial form and motion using each cue's respective reliability, suggesting that in the presence of naturally moving faces, we combine static and dynamic cues in a near-optimal fashion. However, which dynamic aspects exactly contain useful and additional information compared to static faces is still under debate.

### Which dynamic aspects contain information beyond static face information?

An obvious first hypothesis is that the dynamic face advantage is due to a dynamic stimulus providing more samples of the information contained in snapshots of static faces. This was tested using dynamic stimuli in which visual noise masks were inserted between the images making up the stimulus, maintaining the information content of the sequence but eliminating the experience of motion (Ambadar et al., 2005). This manipulation reduced recognition to the level observed with single static frames, thus falsifying this hypothesis. The authors further found that motion enhanced the perception of subtle changes occurring during facial expressions. In a series of experiments, Cunningham and Wallraven (2009) used a similar approach by presenting displays with several static faces as an array or dynamic stimuli with partially or fully randomized frame order. Results again confirmed that dynamic information was coded in the natural deformation of the face over time. Other studies revealed that motion induces a representational momentum during perception of facial expressions which facilitates the detection of changes in the emotion expressed by a face (Yoshikawa and Sato, 2008), that face movement draws attention and increases perception of emotions (Horstmann and Ansorge, 2009) and evokes stronger emotional reactions (Sato and Yoshikawa, 2007). Importantly, most studies investigating the mechanisms underlying the dynamic advantage focused on emotional expressions, ignoring other aspects in which motion contributes less information than form yet still increases performance, such as recognition of facial identity or speech. Therefore, the full picture of what drives the dynamic advantage during face processing is still incomplete.

### Advantages and disadvantages of different kinds of dynamic face stimuli

**Figure 1:**
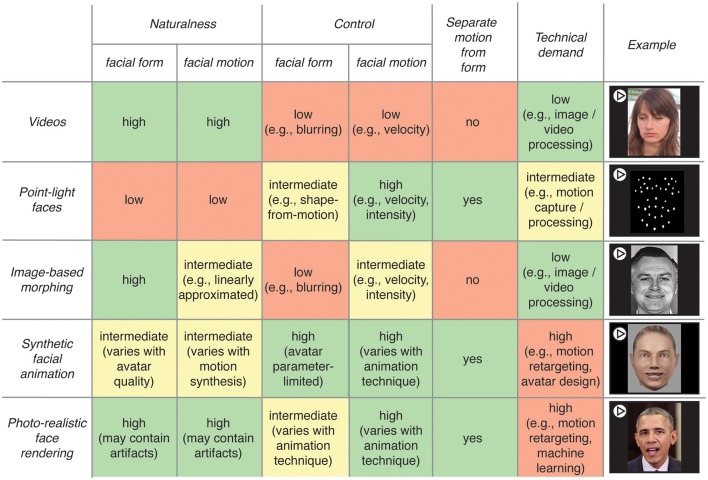
Schematic overview of five different kinds of face stimuli used to investigate dynamic face perception with their respective characteristics. Characteristics include (from left to right): Naturalness of facial form and motion varying between high (e.g., videos), intermediate (e.g., synthetic facial animation), and low (e.g., point-light faces); control of form and motion varying between high (e.g., synthetic facial animation), intermediate (e.g., photo-realistic rendering for form and image-based morphing for motion) or low (e.g., videos); potential for separating motion from form information (e.g., synthetic facial animation); and technical demand varying from low (e.g., videos), to high (e.g., photo-realistic rendering). For ease of comparison, advantages are colored green, intermediate in yellow and disadvantages in orange. Stimuli are listed in no particular order. While the first four kinds of stimuli are commonly used in face perception research, photo-realistic rendering is the most recent advancement and has not yet entered face perception research. [Sources of example stimuli: Videos: (Skerry and Saxe, 2014); Point-light faces: recorded with Optitrack (NaturalPoint, Inc., Corvallis, OR, USA); Image-based morphing: (Ekman and Friesen, 1978); Facial animation: designed in Poser 2012 (SmithMicro, Inc., Watsonville, CA, USA); Photo-realistic rendering: (Suwajanakorn et al., 2017)].

In this section, we give an overview of different types of stimuli that can be used to investigate dynamic face perception. Figure 1 compares five types of stimuli based on the following characteristics: level of naturalness and level of control for form and motion, possibility of manipulating form and motion separately and level of technical demand.

The simplest way to investigate dynamic face perception is to use video recordings of faces (row “Videos” in Figure 1). This has several advantages. First, these stimuli are intuitively more ecologically valid than other types of stimuli since both form and motion are kept natural. Second, videos avoid discrepancies between form and motion naturalness which can reduce perceptual acceptability (e.g., uncanny valley; Mori, 1970). Third, the technical demand is low. Fourth, videos convey spontaneous facial expressions occurring in real-life well, compared to posed facial expressions which tend to be more stereotyped and artificial (Cohn and Schmidt, 2004; Kaulard et al., 2012). Videos have been used to investigate neural representations of emotional valence that generalize across different types of stimuli (Skerry and Saxe, 2014; Kliemann et al., 2018). Other studies have manipulated the order of video frames to investigate the importance of the temporal unfolding of facial expressions (Cunningham and Wallraven, 2009; Reinl and Bartels, 2015; Korolkova, 2018), or the neural sensitivity to natural facial motion dynamics (Schultz and Pilz, 2009; Schultz et al., 2013). While for these research questions, videos of faces achieved a good balance between ecological validity and experimental control, the content of information in such videos is technically challenging to assess (compare “photo-realistic face rendering” below), let alone to parametrically control it.

This control can be achieved using point-light face stimuli (row “Point-light faces” in Figure 1), in which only reflective markers attached to the surface of a moving face are visible. In these stimuli, static form information is typically reduced, while motion information is preserved and fully controllable (i.e., the time courses of marker positions). Studies showed that point-light faces enhance speech comprehension (Rosenblum et al., 1996), that facial expressions can be recognized from such displays (Atkinson et al., 2012) and that subjects are sensitive to the modulation of different properties of point-light faces (Pollick et al., 2003). Despite these valuable findings, one obvious disadvantage of these stimuli is that pure motion and form-from-motion information can hardly be disentangled. For example, what appears like a random point cloud as static display is clearly perceived as a face when in motion. Therefore, the information in facial point-light displays contains both facial motion properties and static face information derived from motion. Taken together, despite their usefulness to investigate perception, point-light stimuli have large drawbacks because they are highly degraded and unnatural and because motion and form-from-motion cues are intermingled.

To address the trade-off between naturalness (e.g., videos of faces) and high degree of control (e.g., point-light faces), an increasing number of studies use image-based morphing techniques (row “image-based morphing” in Figure 1; e.g., by linearly morphing between neutral and peak expression) to create dynamic stimuli. These stimuli represent a compromise between naturalness and experimental control since they allow controlling for motion properties such as intensity or velocity, while the face appears natural. Such stimuli have been used to compare the recognition thresholds for static and dynamic faces (Calvo et al., 2016) or the perception of the intensity of facial expressions (Recio et al., 2014). Despite these useful findings, such stimuli represent solely a coarse linear approximation of natural face motion, which might lead to less accurate emotion recognition than their natural counterparts (Wallraven et al., 2008; Cosker et al., 2010; Korolkova, 2018). Moreover, these stimuli do not allow separating form and motion information, which is necessary to investigate identity-from-motion for example.

To gain full control over form and motion of faces, many studies use synthetic faces animated with facial motion properties (Hill and Johnston, 2001; Knappmeyer et al., 2003; Ku et al., 2005). While such stimuli appear more natural than stimuli based on linear morphing between images (Cosker et al., 2010), perceived naturalness of form and motion varies with the quality of the synthetic faces and the motion used for animation (Wallraven et al., 2008). One way to generate such stimuli is to use recorded marker-based motion data (see “Point-light faces” above) from actors performing facial actions, and to map these to synthetic faces (e.g., Hill and Johnston, 2001; Knappmeyer et al., 2003). Drawbacks are the difficulty to map specific markers to face regions, and artifacts resulting from shape differences between recorded and target faces. Further, while the resulting animations can closely approximate natural expressions, systematically manipulating and interpreting the underlying motion properties remains complex. To address this challenge, complex and detailed movements can be created using a common coding scheme for facial motion called Facial Action Coding System (FACS; Ekman and Friesen, 1978). This system uses a number of discrete ‘face movements’ - termed Action Units - to describe the basic components of most facial actions. Importantly, the motion properties of each Action Unit can be semantically described (e.g., eyebrow raising) and modified separately to induce systematic local changes in facial motion (Jack et al., 2012; Yu et al., 2012). Synthetic faces can be animated based on Action Unit time courses extracted from real motion-capture data (Curio et al., 2006) or synthesized in the absence of actor data (Roesch et al., 2010; Yu et al., 2012). Overall, such animations allow meaningful interpretation, quantification as well as systematic manipulation of motion properties, with full control over form. The main shortcomings are the high technical demands to create these stimuli, and the fact that the faces are synthetic.

Major advancements in the development of face tracking and animations have recently been made. In particular, it is now possible to animate faces in a photo-realistic fashion (see row “Photo-realistic rendering” in Figure 1). These recent developments bear potential for face perception research. First, new developments reduce the technical demands of recording facial movements allowing markerless tracking by using for example depth sensors (e.g., Walder et al., 2009; Girges et al., 2015), automated landmark detection (Korolkova, 2018), or simply RGB channels in videos (Thies et al., 2016). Second, recent facial animation and machine learning advancements (e.g., deep learning) now allow creating naturalistic dynamic face stimuli indistinguishable from real videos (e.g., Thies et al., 2016; Suwajanakorn et al., 2017). While these technologies have hardly entered face perception research to date, we believe that a novel and promising approach will consist in collaborating with computer vision labs to address open questions in face perception.

## Conclusion and future directions

In this review, we discuss the usefulness of dynamic faces for face perception studies, review the conditions under which dynamic advantages arise, and compare different kinds of stimuli used to investigate dynamic face processing. The finding that the dynamic advantage was less pronounced when other cues convey similar or more reliable information fits the view that the brain constantly integrates sensory cues (e.g., dynamic and static) based on their respective reliabilities to achieve robust perception. While such an integration mechanism was shown for identity recognition (Dobs et al., 2017), the mechanisms underlying the perception of other facial aspects (e.g., gender, age or health) still need to be unraveled. Moreover, most studies investigated faces presented alone; yet when interpreting the mood or intention of a vis-à-vis in daily life, humans do not take solely facial form and movements into account, but also gaze motion, voice, speech, so as motion of the head or even the whole body (e.g., Van den Stock et al., 2007; Dukes et al., 2017). To better understand these aspects of face perception, future face perception studies would benefit from the use of models of cue integration as well as dynamic and multisensory face stimuli (e.g., gaze, voice).

What kind of dynamic stimulus is appropriate to study which aspect of face perception? Each of the dynamic stimuli reviewed here has specific advantages and disadvantages; it is thus difficult to make general suggestions. Findings showed that the face perception system is highly sensitive to natural facial motion, which supports the use of dynamic face stimuli based on real face motion. However, to our knowledge, a systematic investigation of differences in processing faces across different types of stimuli (e.g., synthetic faces vs. videos) is still lacking, and thus the generalizability of findings from studies using synthetic or point-light faces is still unclear and should be addressed in future studies.

Furthermore, it is still unclear which motion properties are used by the face perception system. Advances were made in the realm of dynamic expressions of emotions, but more controlled studies and paradigms are needed. Synthetic facial animations or even photo-realistic face rendering providing high control over form and motion are promising candidate stimuli to investigate these questions. For example, using synthetic facial animations and a reverse correlation technique, Jack et al. (2012) revealed cultural differences in perception of emotions from dynamic stimuli and identified the motion properties contributing to these differences. Similar techniques might help to characterize which properties convey idiosyncratic facial movements for example, and the dynamic advantage in general.

Finally, a major remaining question addresses the representation of facial motion in the human face perception system. How many dimensions are used to encode the full space of facial motions, and what are these dimensions? Recent evidence suggests that a small number of dimensions are sufficient (Dobs et al., 2014; Chiovetto et al., 2018) but more studies based on larger data sets are needed. If a set of basic components can be characterized, can we identify behavioral and neural correlates of a facial motion space, similar to what is known as face space for static faces (Valentine, 2001; Leopold et al., 2006; Chang and Tsao, 2017)?

## Author contributions

KD, IB, and JS designed the concept of the article, reviewed the literature and wrote the article.

### Conflict of interest statement

The authors declare that the research was conducted in the absence of any commercial or financial relationships that could be construed as a potential conflict of interest.
